# Anti-angiogenic therapy for cancer: current progress, unresolved questions and future directions

**DOI:** 10.1007/s10456-014-9420-y

**Published:** 2014-01-31

**Authors:** Naveen S. Vasudev, Andrew R. Reynolds

**Affiliations:** 1 Tumour Biology Team, Breakthrough Breast Cancer Research Centre, The Institute of Cancer Research, Fulham Road, London, SW3 6JB UK; 2 Present Address: Cancer Research UK Centre, Leeds Institute of Cancer and Pathology, St James’s University Hospital, Beckett Street, Leeds, LS9 7TF UK

**Keywords:** VEGF, Angiogenesis, Metastasis, Resistance, Microenvironment, Personalised medicine

## Abstract

Tumours require a vascular supply to grow and can achieve this via the expression of pro-angiogenic growth factors, including members of the vascular endothelial growth factor (VEGF) family of ligands. Since one or more of the VEGF ligand family is overexpressed in most solid cancers, there was great optimism that inhibition of the VEGF pathway would represent an effective anti-angiogenic therapy for most tumour types. Encouragingly, VEGF pathway targeted drugs such as bevacizumab, sunitinib and aflibercept have shown activity in certain settings. However, inhibition of VEGF signalling is not effective in all cancers, prompting the need to further understand how the vasculature can be effectively targeted in tumours. Here we present a succinct review of the progress with VEGF-targeted therapy and the unresolved questions that exist in the field: including its use in different disease stages (metastatic, adjuvant, neoadjuvant), interactions with chemotherapy, duration and scheduling of therapy, potential predictive biomarkers and proposed mechanisms of resistance, including paradoxical effects such as enhanced tumour aggressiveness. In terms of future directions, we discuss the need to delineate further the complexities of tumour vascularisation if we are to develop more effective and personalised anti-angiogenic therapies.

## Introduction

**Fig. 1:**
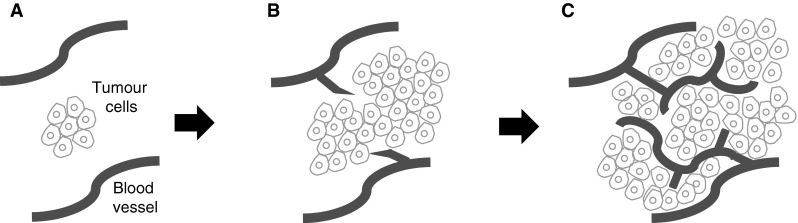
The role of sprouting angiogenesis in tumour growth. Early observations on the growth of tumours supported the following model for how tumours obtain a vascular supply. **a** When a tumour mass is small, it can obtain oxygen and nutrients from existing local blood vessels. **b** As the tumour grows beyond the capacity of local blood vessels, soluble pro-angiogenic factors are released which promote the sprouting of new vessels from local existing blood vessels (sprouting angiogenesis). **c** These vessels provide a blood supply for the tumour and this is required in order for the tumour to grow beyond 2–3 mm in size

**Fig. 2:**
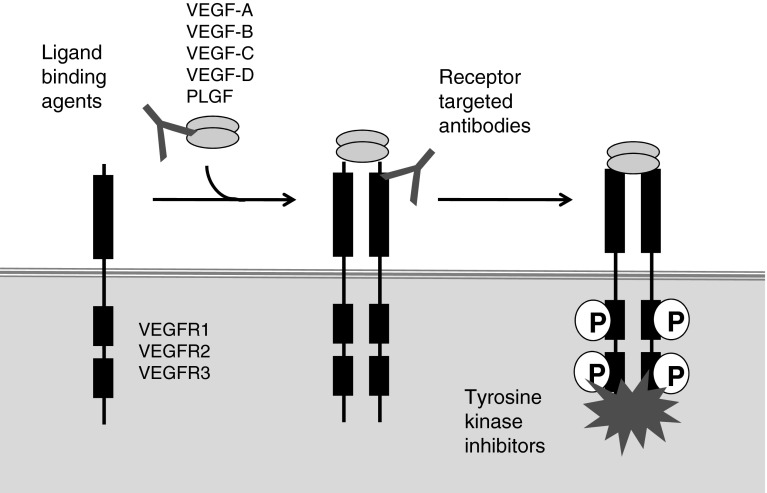
VEGF-targeted agents. The VEGF signalling system in mammals is complex and consists of five related ligands, VEGF-A, VEGF-B, VEGF-C, VEGF-D and PLGF that bind with different specificities to three receptor tyrosine kinases, VEGFR1, VEGFR2 and VEGFR3. The biology of these interactions has been extensively reviewed [231, 233]. Shown is a highly simplified diagram designed to illustrate the three major classes of agent that target this signalling system: (a) ligand binding agents that block the binding of VEGF ligands to receptors (e.g. bevacizumab which binds to VEGF-A alone and aflibercept which binds to VEGF-A, VEGF-B and PLGF), (b) antibodies that block signalling through VEGF receptors (e.g. ramucirumab which binds to VEGFR2) and (c) tyrosine kinase inhibitors which block the kinase activity of VEGFR1, VEGFR2 and VEGFR3 (e.g. sorafenib, sunitinib, pazopanib). Note that these tyrosine kinase inhibitors can also can inhibit the kinase activity of some other receptor tyrosine kinases, including platelet derived growth factor receptors (PDGFRs), c-Kit and fms-related tyrosine kinase (FLT3) [233]

The concept of ‘anti-angiogenic therapy’ arose from the seminal observations of Judah Folkman and colleagues. Pre-clinical studies showed that tumours induce the sprouting of new vessels from the surrounding vasculature (sprouting angiogenesis) and that this process is vital for the growth of tumours beyond 2–3 mm in size (Fig. 1). It was therefore proposed that inhibition of sprouting angiogenesis could suppress tumour growth in humans [1]. Further studies established that (a) vascular endothelial growth factor-A (VEGF) is a key driver of sprouting angiogenesis, (b) VEGF is overexpressed in most solid cancers, and (c) inhibition of VEGF can suppress tumour growth in animal models [2–4]. Based on these observations, numerous therapies have been developed that target angiogenesis by blocking the VEGF signalling pathway (Fig. 2). The biology of VEGF signalling, angiogenesis and the principles upon which anti-angiogenic therapy is based have been extensively reviewed [2, 5–8]. Here we review the progress of VEGF-targeted therapies in the clinic (see also Table 1), discuss the current questions and controversies that exist in the field and propose routes to more effective and personalised anti-angiogenic therapy.

**Table 1:** Randomised trials of anti-angiogenic agents cited in this article

Indication	Treatment	Trial identifier and citation	Outcome
*Breast cancer*			
Metastatic 1st line	Paclitaxel ± bevacizumab	E2100 [40]	Improvement in PFS not OS
	Docetaxel ± bevacizumab (HER-2 negative population)	AVADO [41]	Improvement in PFS, OS NA
	Capecitabine, taxane or anthracycline ± bevacizumab (HER-2 negative population)	RIBBON-1 [42]	Improvement in PFS but not in OS
	Docetaxel and trastuzumab ± bevacizumab (HER-2 positive population)	AVEREL [104]	No improvement in PFS, OS NA
	Docetaxel ± sunitinib (HER-2 negative population)	Sun 1064 [45]	No improvement in PFS or OS
	Paclitaxel ± bevacizumab or sunitinib (HER-2 negative population)	SUN 1094 [46]	Inferior PFS for sunitinib arm
Metastatic 2nd line and beyond	Capecitabine ± bevacizumab	AVF2119 [39]	No improvement in PFS or OS
	Capecitabine, taxane, gemcitabine, or vinorelbine ± bevacizumab (HER-2 negative population)	RIBBON-2 [43]	Improvement in PFS but not in OS
	Capecitabine ± sunitinib	NCT00435409 [44]	No improvement in PFS or OS
	Capecitabine vs. sunitinib (HER-2 negative population)	SUN 1107 [47]	Inferior PFS and OS for sunitinib arm
Adjuvant	Anthracycline, taxane or both ± bevacizumab (triple negative population)	BEATRICE [58]	No improvement in DFS, OS NA
Neo-adjuvant	Doxorubicin/docetaxel/cyclophosphamide ± bevacizumab	NCT00408408 [63]	Improvement in pathological complete response rate (primary endpoint)
	Epirubicin/docetaxel/Cyclophosphamide ± bevacizumab (HER-2 negative population)	NCT00567554 [64]	Improvement in pathological complete response rate (primary endpoint)
*Colorectal cancer*			
Metastatic 1st line	FOLFIRI ± bevacizumab	AVF2107 [19]	Improvement in OS and PFS
	FOLFOX or XELOX ± bevacizumab	NO16966 [21]	Improvement in OS and PFS
	Capecitabine ± bevacizumab	AVEX [22]	Improvement in PFS, OS NA
	FOLFIRI ± sunitinib	SUN1122 [28]	No improvement in PFS
	FOLFOX ± vatalanib	CONFIRM 1 [29]	No improvement in PFS or OS
Metastatic 2nd line and beyond	FOLFOX ± bevacizumab	E3200 [20]	Improvement in OS and PFS
	FOLFOX ± vatalanib	CONFIRM 2 [30]	Improvement in PFS but not OS
	FOLFIRI ± aflibercept	VELOUR [27]	Improvement in OS and PFS
	Regorafenib versus placebo	CORRECT [31]	Improvement in OS
Continuation beyond progression	Chemotherapy ± bevacizumab	ML18 147 [92]	Improvement in OS
Adjuvant	FOLFOX ± bevacizumab	NSABP C-08 [56]	No improvement in OS
	FOLFOX or XELOX ± bevacizumab	AVANT [57]	No improvement in OS
*Hepatocellular carcinoma*			
Metastatic 1st line	Sorafenib versus placebo	NCT00105443 [17]	Improvement in PFS and OS
	Brivanib versus sorafenib	BRISK-FL [145]	OS non-inferiority end-point for brivanib versus sorafenib not met
Metastatic 2nd line	Brivanib versus placebo	BRISK-PS [146]	Improvement in PFS but not OS
*Melanoma*			
Metastatic 1st line	Paclitaxel/carboplatin ± bevacizumab	BEAM*** [48]	No improvement in PFS or OS
	Paclitaxel/carboplatin ± sorafenib	NCT00110019 [49]	No improvement in PFS or OS
Metastatic 2nd line	Paclitaxel/carboplatin ± sorafenib	NCT00111007 [50]	No improvement in PFS or OS
NSCLC*			
Metastatic 1st line	Paclitaxel/carboplatin ± bevacizumab	NCT00021060 [32]	Improvement in PFS and OS
	Cisplatin/gemcitabine ± bevacizumab	AVAiL [33]	Improvement in PFS but not OS
*Ovarian cancer*			
Metastatic 1st line	Paclitaxel/carboplatin ± bevacizumab	ICON-7 [36]	Improvement in PFS, OS NA
	Paclitaxel/carboplatin ± bevacizumab	GOG218 [37]	Improvement in PFS, OS confounded by cross-over
Metastatic 2nd line	Gemcitabine/carboplatin ± bevacizumab	OCEANS [38]	Improvement in PFS but not OS
*Pancreatic cancer*			
Metastatic 1st line	Gemcitabine ± bevacizumab	CALGB 80303 [51]	No improvement in PFS or OS
*PNET*			
Metastatic 1st line	Sunitinib versus placebo	NCT00428597 [18]	Improvement in PFS, OS NA
Prostate cancer**			
Metastatic 1st line	Docetaxel/prednisone ± bevacizumab	CALGB 90401 [52]	Improvement in PFS but not OS
	Docetaxel/prednisone ± aflibercept	VENICE [53]	No improvement in PFS or OS
*Renal cancer*			
Metastatic 1st line	Sorafenib versus placebo	TARGET [9]	Improvement in PFS and OS
	Sunitinib versus interferon-alpha	NCT00098657 [11]	Improvement in PFS and OS
	Pazopanib versus placebo	NCT00334282 [13]	Improvement in PFS, OS confounded by cross-over
	Sunitinib versus pazopanib	COMPARZ [15]	PFS and OS were similar
Metastatic 2nd line	Axitinib versus sorafenib	AXIS [16]	Improvement in favour of axitinib for PFS but not OS

*DFS* disease-free survival, *FOLFIRI* 5-FU, leucovorin and irinotecan, *FOLFOX* 5-FU, leucovorin and oxaliplatin, *HER-2* human epidermal growth factor receptor-2, *NA* not available (pending, unknown or not reported), *NSCLC* non-small cell lung cancer, *OS* overall survival, *PNET* pancreatic neuroendocrine tumour, *PFS* progression-free survival

* Non-squamous NSCLC only; ** castration resistant; *** randomised phase II study

## Anti-angiogenic therapy in the metastatic setting

Since angiogenesis is deemed necessary for the growth of metastases in all sites of the body, it is assumed that anti-angiogenic therapy should be of benefit for patients with metastatic disease. However, variable results have been obtained across different cancer types, suggesting that whilst the metastases of certain cancers are sensitive to this form of therapy, the metastases of others are not. Tyrosine kinase inhibitors (TKIs), designed to inhibit VEGF receptor signalling (Fig. 2), have demonstrated single-agent activity in several indications. In metastatic renal cell carcinoma (mRCC) these agents have proven highly successful, with four drugs now FDA approved in this setting, namely sorafenib, sunitinib, pazopanib and axitinib. Sorafenib was the first TKI to demonstrate activity in mRCC, in a placebo-controlled phase III randomised trial of patients who had progressed on previous cytokine therapy [9]. Progression free survival (PFS) was almost doubled (5.5 vs. 2.8 months) and an improvement in overall survival (OS) was observed when placebo-treated patients crossing over to sorafenib were excluded from the analysis [10]. A subsequent study comparing single agent sunitinib with interferon-α in mRCC patients (that were naïve to treatment) demonstrated a significant improvement in PFS in the sunitinib arm (11 vs. 5 months) [11]. Improvement in OS was observed in the sunitinib arm (26.4 vs 21.8 months) and in a subset-analysis of patients who did not receive any post-study cancer treatment, improvement in OS was even more marked (28.1 vs. 14.1 months) [12]. Single agent pazopanib compared with placebo was subsequently shown to extend PFS in mRCC in the first-line setting (11.1 vs. 2.8 months), but extensive crossover from placebo to pazopanib confounded the final OS analysis [13, 14]. A recent phase III trial comparing sunitinib with pazopanib has demonstrated that both drugs have similar efficacy [15] and single agent therapy with either drug is now recommended as standard of care in the first-line in mRCC. Axitinib, a more recently developed TKI, has shown efficacy in the second-line setting in patients that progressed on first-line TKI therapy [16] and is now recommended for mRCC in this setting.

TKIs have also shown single agent activity in advanced hepatocellular carcinoma and advanced pancreatic neuroendocrine tumours (PNET). In hepatocellular carcinoma, sorafenib improved OS from 7.9 to 10.7 months versus placebo in a randomised phase III study, leading to its FDA approval in 2007 [17]. Sunitinib is FDA-approved for the treatment of PNET based on the results of a randomised placebo-controlled study that demonstrated doubling of PFS from 5.5 months in the control arm to 11.4 months in the sunitinib arm, although the OS analysis was confounded by cross-over of patients from the control arm to the sunitinib arm [18].

Bevacizumab, a humanised monoclonal antibody that binds specifically to VEGF-A alone, has shown efficacy in several indications in the metastatic setting. The first phase III trial published demonstrating the efficacy of an anti-angiogenic agent in the clinic was in metastatic colorectal cancer (mCRC), where the combination of chemotherapy with bevacizumab was shown to result in superior PFS (10.6 vs. 6.2 months) and OS (23 vs. 15.3 months) compared to the chemotherapy only arm [19]. Based on these data, bevacizumab was approved for the treatment of mCRC when given in combination with chemotherapy. Subsequent phase III studies have also demonstrated a beneficial effect of adding bevacizumab to chemotherapy in mCRC [20–22]. Additional evidence for the efficacy of anti-angiogenic therapy in colorectal cancer comes from a study of aflibercept, a novel fusion protein that binds to three VEGF family ligands: VEGF-A, VEGF-B and placental growth factor (PLGF). By targeting VEGF-B and PLGF, which are also implicated in angiogenesis and/or the survival of newly formed vessels, aflibercept may have additional anti-angiogenic effects beyond targeting VEGF-A alone [23–26]. Adding aflibercept to chemotherapy was shown to extend PFS and OS compared to chemotherapy alone in metastatic colorectal cancer [27]. Moreover, a striking separation of the survival curves was observed in this study, with 2-year survival significantly increased in the aflibercept arm compared to the control arm (28.0 vs. 18.7 %) [27]. Based on these data, aflibercept was recently approved for the treatment of mCRC when given in combination with chemotherapy.

Curiously, despite the benefit observed when bevacizumab or aflibercept are combined with chemotherapy in mCRC, efforts to combine anti-angiogenic TKIs with chemotherapy in mCRC have so far proven disappointing in terms of improving OS [28–30]. However, single agent treatment with the TKI regorafenib was recently reported to extend OS compared to placebo in mCRC patients who had previously progressed on standard therapies [31]. Regorafenib is now approved for the treatment of mCRC in this setting.

In non-squamous non-small cell lung cancer (NSCLC), two phase III trials have shown an improvement in PFS for the addition of bevacizumab to chemotherapy [32–34] although only one study reported an improvement in OS [32]. A recent meta-analysis, combining data from these two phase III studies (plus data from two phase II studies) including >2,000 patients, concluded a small but significant improvement in OS of 4 % at 1 year [35].

In the first-line treatment of ovarian cancer, two pivotal studies (ICON-7 and GOG218) have been reported examining the addition of bevacizumab to chemotherapy [36, 37]. Both studies reported a significant improvement in PFS of between 2.4 and 3.8 months. OS data were not significant in the GOG218 study (but were confounded due to cross-over) and OS data are still awaited for the ICON7 study. However, in ICON7, an improvement in overall survival with bevacizumab was observed in the high-risk group compared to chemotherapy alone (36.6 vs. 28.8 months). In relapsed ovarian cancer, the addition of bevacizumab to chemotherapy has demonstrated a significant improvement in PFS, although this has not translated into an OS benefit [38].

In contrast to these promising data, there are several notable examples of metastatic cancers where anti-angiogenic agents have consistently failed to make a significant impact on overall survival, including breast, melanoma, pancreatic and prostate. The history of anti-angiogenic therapy in the treatment of metastatic breast cancer is of significant interest. In 2005, the AVF2119 phase III study demonstrated that the addition of bevacizumab to capecitabine did not result in extension of either PFS or OS in metastatic breast cancer [39]. However, in 2007, the E2100 phase III study demonstrated that the addition of bevacizumab to paclitaxel resulted in extension of PFS (11.8 vs. 5.9 months), but not OS, in metastatic breast cancer [40]. On the basis of these data, the FDA granted the accelerated approval of bevacizumab in combination with paclitaxel for the treatment of HER2-negative metastatic breast cancer. Three further phase III trials of bevacizumab in combination with chemotherapy in HER2-negative metastatic breast cancer (AVADO, RIBBON-1 and RIBBON-2) demonstrated an extension of PFS, but no effect on OS, when compared to chemotherapy alone [41–43]. In 2010, the FDA concluded that the results of these studies failed to provide evidence that bevacizumab could prolong survival in metastatic breast cancer. As a consequence of this, in 2010 the FDA withdrew its approval for bevacizumab in this indication. In addition to this, disappointing results have also been observed with TKIs in breast cancer. Three phase III studies examining the addition of sunitinib to chemotherapy [44–46], and one comparing single agent sunitinib versus chemotherapy [47], all failed to demonstrate improvement in PFS or OS.

Studies in melanoma assessing the benefit of adding either bevacizumab [48] or sorafenib [49, 50] to chemotherapy in the first- and/or second-line setting have all failed to reach their primary efficacy end-point of PFS. In adenocarcinoma of the pancreas, the addition of bevacizumab to chemotherapy in a phase III randomised trial failed to improve PFS [51]. In men with castrate-resistant prostate cancer, the addition of bevacizumab [52], or aflibercept [53], to chemotherapy have failed to improve OS in comparison to chemotherapy alone.

The precise explanation as to why conventional anti-angiogenic agents show efficacy in some metastatic cancers, and not others, is currently unknown [54]. Conceivably, important differences in the biology of these cancers may underlie the contrasting results seen with this therapeutic approach across different cancers.

## Anti-angiogenic therapy in the adjuvant setting

The use of anti-angiogenic agents in the adjuvant setting is based on the principle that, after surgical removal of the primary tumour, inhibition of angiogenesis may prevent local relapse or the growth of micrometastatic tumours. Two phase III trials in the adjuvant setting (NSABP C-08 and AVANT) were designed to compare overall survival in colorectal cancer patients treated with chemotherapy alone for 6 months in one arm and chemotherapy plus bevacizumab for 6 months (followed by 6 months bevacizumab maintenance therapy) in the second arm. In both trials, an analysis performed after 1 year demonstrated improved PFS in the bevacizumab arm. However, no significant difference in OS was observed between treatment arms when assessed at 3 or 5 years [55–57]. In addition to these data, recently disclosed findings from the BEATRICE trial show that adjuvant bevacizumab failed to improve disease free survival in triple negative breast cancer patients at 3 years [58].

Given the efficacy demonstrated for bevacizumab in metastatic colorectal cancer, the poor results achieved in the adjuvant setting are clearly disappointing. The results suggest that, even in a disease where anti-angiogenic therapy is shown to be effective in the metastatic setting, the same may not be true when used in the adjuvant setting. However, this situation is not unique to bevacizumab, because it has been reported for other agents in colorectal cancer. In colorectal cancer, for many years the quest for successful adjuvant therapies has followed a simple and reliable path. Drugs such as 5-FU, oxaliplatin and capecitabine were first shown to be effective in the metastatic setting, which was followed by successful trials in the adjuvant setting [59–61]. However, there are now three notable exceptions that have not followed this path: irinotecan, cetuximab and bevacizumab have all shown efficacy in the metastatic setting, but failed in the adjuvant setting in colorectal cancer [56, 57, 59, 62]. The reasons that underlie these discrepant results are currently unknown. However, it seems most likely that the biology of micrometastases can be very different to the biology of established metastatic disease and that this has important consequences for therapy response.

## Anti-angiogenic therapy in the neoadjuvant setting

Theoretically, there may be several advantages to using anti-angiogenic therapy in the neoadjuvant setting. Firstly, it might be used to downsize a tumour in order to convert a non-resectable lesion to one that is potentially resectable. Secondly, it might be used to downstage the disease to reduce the chance of local relapse or metastasis. Two large randomised trials recently reported on the efficacy of bevacizumab plus chemotherapy as a neoadjuvant therapy for primary breast cancer compared to neoadjuvant chemotherapy alone [63, 64]. Both used pathological complete response (pCR) as the endpoint. Although a significant increase in the rate of pCR was observed, the absolute increase in response rate was small (3.5 and 6.3 %, respectively). Moreover, subgroup analysis revealed contradictory findings, with one study reporting greater benefit in women with hormone receptor negative (triple negative) disease [64] and the other study suggesting that women with hormone receptor positive cancer were more likely to benefit [63]. It is as yet unclear whether any survival benefit will be associated with the use of bevacizumab in this setting because there is currently no mature data.

In CRC, surgical resection of liver metastases is potentially curative and has significantly improved overall survival in this setting [65]. Although only a fraction of patients are resectable at presentation the use of neoadjuvant chemotherapy to convert unresectable metastases to potentially resectable metastases has lead to improvements in resection rates and is a recommended practice [66]. Interestingly, there is evidence to suggest that combination of bevacizumab with chemotherapy may also be an effective conversion therapy for CRC liver metastasis [67–69]. However, a randomised trial directly comparing the efficacy of chemotherapy versus chemotherapy combined with an anti-angiogenic agent has not been undertaken in this setting.

## Interactions with chemotherapy

In most settings, with the exception of ovarian cancer where single agent activity for bevacizumab has been observed [70], anti-angiogenic agents such as bevacizumab and aflibercept have only shown significant activity when they are combined with cytotoxic chemotherapy [19, 27]. How can this be explained? For some time, the prevailing explanation for this effect has been the concept of ‘vascular normalisation.’ Tumour vessels are known to be leaky and dysfunctional, leading to increased interstitial fluid pressure, which may in turn impede the delivery of chemotherapy [71, 72]. Preclinical studies showed that suppression of VEGF signalling can lead to improvements in tumour vessel function (vascular normalisation), and in turn, this was proposed to mediate increased delivery of chemotherapy to tumours [71, 72]. Therefore, a widely held conception is that bevacizumab ‘works’ in the clinic because it improves the delivery of co-administered chemotherapy. However, the clinical relevance of this phenomenon for chemotherapy delivery in patients is still unclear. For example, the addition of bevacizumab to chemotherapy would be expected to lead to improvements across the board in all settings, but this is not the case. Moreover, a recent study reported the opposite relationship i.e. bevacizumab led to a sustained decrease in the delivery of chemotherapy in NSCLC patients [73].

At this point it should be noted that pharmacological induction of vessel normalisation may have additional therapeutic effects in cancer beyond control of chemotherapy delivery. For example, in glioblastoma patients, vessel normalisation induced by VEGF-targeted therapy may prolong survival due to alternative mechanisms involving oedema control or improved tumour oxygenation [74, 75]. Despite these facts, it is still not clear why agents like bevacizumab and aflibercept show greater activity when they are combined with chemotherapy. Any number of alternative mechanisms could underlie this activity. For example, an alternative explanation is that anti-angiogenic drugs prevent the rebound in tumour growth that may occur during breaks in chemotherapy [76] or counteract the ability of chemotherapy to promote tumour invasion [77]. Importantly, in contrast to bevacizumab, TKIs generally show single agent activity and so any mechanistic explanation for the synergy between VEGF-targeted agents and chemotherapy must account for this unexplained dichotomy. A recent study, which examined data from both clinical samples and preclinical models, provided intriguing evidence that this dichotomy may stem from intrinsic differences in the stromal component of different cancers [78]. They provided evidence that, in cancers that are more responsive to bevacizumab when it is combined with chemotherapy (e.g. mCRC, NSCLC), the vasculature has a stromal-vessel phenotype, where the vessels are surrounded by a well-developed stroma. In contrast, cancers that are responsive to single agent TKIs (e.g. mRCC, PNET) have a tumour-vessel phenotype, where the vessels sit closer to the tumour cells without a well-developed intervening stromal component. Although the molecular mechanisms were not uncovered, these data do suggest that an interaction between multiple stromal components influences the response to anti-angiogenic therapy.

Therefore, our understanding of why TKIs work as single agents and why VEGF-targeted agents synergise with chemotherapy in patients is still incomplete. A further unresolved question is whether certain types of chemotherapy may work better with bevacizumab than others. Several on-going phase III studies in advanced breast cancer will address the efficacy of bevacizumab when combined with different chemotherapies or with other targeted agents [79, 80]. However, further studies that elaborate on the mechanistic basis for the interaction of chemotherapy with VEGF-targeted therapies are urgently needed.

## Toxicity

It was assumed that because angiogenesis is a relatively rare process in the adult, VEGF-targeted therapies would be toxicity free. However, clinical experience reveals a number of adverse events associated with these agents, including hypertension, proteinuria, impaired wound healing, gastrointestinal perforation, haemorrhage, thrombosis, reversible posterior leukoencephalopathy, cardiac toxicity and endocrine dysfunction, which have been extensively reviewed [81, 82]. Although some of these side effects can be managed in a routine fashion, excessive toxicity may necessitate the use of treatment breaks, dose reductions or even treatment cessation, which may limit therapeutic efficacy. However, it has also been proposed that certain side effects could be used as a predictive biomarker for efficacy. Several studies have demonstrated a link between the development of hypertension and longer PFS/and or OS in patients treated with anti-angiogenic agents [83–86]. It has been suggested that, if this association can be validated prospectively, then assessment of hypertension early in treatment might be used to stratify patients likely to benefit from anti-angiogenic therapy versus those that might be transferred to an alternative therapy.

## Duration and scheduling of therapy

Preclinical and clinical work shows that when VEGF-targeted therapy is discontinued, the tumour vasculature can become rapidly re-established [87, 88]. Conceivably, this could lead to tumour re-growth when therapy is withdrawn. Indeed, there are reports of tumour re-growth during planned treatment breaks in anti-angiogenic therapy [89, 90]. These data suggest that prolonged use of VEGF-targeted therapy may be necessary to achieve maximal therapeutic benefit. In support of this, an observational study, which analysed data from 1,445 patients treated with bevacizumab, showed that continuation of bevacizumab treatment beyond progression was indeed associated with greater benefit in terms of overall survival [91]. This observation was recently validated prospectively in mCRC in the ML18 147 trial [92].

Another interesting observation is that acquired resistance to anti-angiogenic therapy may in some cases be a transient phenomenon. Following the development of resistance to one VEGF-targeted agent, mRCC patients have been transferred to a second course of VEGF-targeted therapy. Surprisingly, a proportion of these re-challenged patients respond again to therapy [93–95]. Moreover, the benefit that is achieved upon re-challenge can be proportional to the length of time that elapses between therapy [96]. These data suggest that resistance to VEGF-targeted therapy can sometimes be a reversible phenomenon [97]. There are some interesting parallels between these data and preclinical studies also showing that resistance to VEGF-targeted therapy can be reversible [98, 99]. Based on these data, it seems possible that the incorporation of strategic treatment breaks might help to ‘reset’ tumour resistance and avoid the onset of acquired resistance. However, this idea has yet to be formally proved in the clinic.

## Predictive biomarkers

Given the variable results obtained with anti-angiogenic agents in the clinic, there is a need to distinguish which patients are likely to benefit from this form of therapy from those patients that will not. This entails the development of predictive biomarkers that are capable of predicting response or outcome [100–102]. However, despite intensive efforts, there are currently no validated biomarkers for selecting these patients. Many types of predictive biomarkers have been investigated, including hypertension, circulating markers, germline single nucleotide polymorphisms (SNPs), in situ markers in tumour material and functional imaging. This area has been extensively reviewed [101, 102] and we will cover here only some recent developments in circulating markers, SNPs and imaging.

### Circulating markers

Historically speaking, studies examining baseline-circulating levels of angiogenesis-related factors, such as VEGF, have yielded disappointing and contradictory findings, often providing prognostic rather than predictive information [10, 101–103]. However, recent studies, based on prospective, robust sample sets collected within clinical trials are now starting to show more consistent results. For example, a correlation between high circulating levels of VEGF-A and survival benefit in metastatic breast and gastric cancer patients treated with bevacizumab has been reported [104–106]. A large phase III trial (MERiDIAN) will prospectively test the utility of high circulating VEGF-A levels as a potential biomarker of response to bevacizumab in HER2-negative metastatic breast cancer [105].

Biomarker signatures, composed of multiple circulating factors, may also have potential value as predictive biomarkers. In pazopanib-treated mRCC patients for example, circulating levels of six serum cytokines and angiogenesis factors (CAF) (HGF, interleukin 6, interleukin 8, osteopontin, VEGF, and TIMP1) were able to identify a sub-set of patients that derived significantly greater overall survival benefit from treatment [107]. Moreover, a serum-based protein signature composed of mesothelin, FLT4, AGP and CA125 has recently been shown to identify patients with ovarian cancer more likely to benefit from bevacizumab [108].

However, there are several challenges associated with taking circulating factors forward as a prospective marker. Firstly, measurement of circulating markers can be difficult to standardise across centres, due to technical issues associated with sample handling [109]. Secondly, deciding on a predefined cut-off for high versus low levels of circulating factors is challenging because it may vary with geography and disease setting [109].

### SNPs

Baseline predictive markers that are binary in nature (i.e. a mutation or gene amplification) are attractive because they may be easier to measure and apply prospectively than biomarkers based on the measurement of circulating factors. A large study that examined data from two phase III trials of bevacizumab in metastatic pancreatic adenocarcinoma (AViTA) and mRCC (AVOREN) recently reported that a SNP in VEGFR1 was significantly associated with poor outcome in patients treated with bevacizumab [110]. The same SNP has subsequently been associated with poor outcome in mRCC patients treated with sunitinib [111]. Fine mapping of this SNP to tyrosine 1,213 of VEGFR1 shows that mutation at this site leads to increased expression and signalling of VEGFR1, providing a plausible explanation as to why VEGF-targeted therapy is less effective in patients bearing this SNP [110]. Therefore, this work identifies a negative biomarker that might be used prospectively to exclude patients who are less likely to benefit from VEGF-targeted therapy.

### Imaging

Functional imaging of the tumour vasculature, using CT, MRI or PET, is a potentially attractive approach for predicting response and outcome, as reviewed in [112]. Imaging permits inspection of various parameters, such as tumour morphology and blood flow, which may provide important predictive information. There are studies showing that baseline features of tumours, such as the level of vascular perfusion, can predict response or outcome in patients treated with anti-angiogenic agents. For example, at least 4 published studies demonstrate that a high level of vascular perfusion predicts for response or outcome in mRCC patients treated with TKIs [113–116]. Early changes in vascular characteristics detected on imaging after the initiation of therapy have also been shown to correlate with response or outcome. For example, many studies performed in mRCC patients treated with TKIs show that a reduction in vascular perfusion on therapy provides extra predictive information regarding response or outcome than using criteria based on change in lesion size alone [112, 117–123]. Moreover, in patients with colorectal liver metastases treated with bevacizumab and chemotherapy, changes in tumour morphology on CT were shown to associate more significantly with overall survival than the use of RECIST criteria [124]. Although these studies suggest a promising role for imaging as a predictive marker in certain settings, many challenges remain. For example, we have an incomplete understanding of how features detected on imaging correlate with the underlying tumour biology [112]. Also, methodologies used to assess imaging biomarkers vary considerably between studies and require standardisation for their prospective application across multiple study centres [112].

Therefore, biomarkers that predict response or outcome for VEGF-targeted therapy are emerging, but they require further standardisation and validation before they are incorporated into clinical practice.

## Mechanisms of response and resistance to VEGF-targeted therapy

Resistance to anti-angiogenic therapy is a prominent issue that likely explains the variable results obtained in the clinic with this approach. Resistance can broadly be classified into intrinsic resistance (where tumours fail to respond from the outset of treatment) and acquired resistance (where tumours initially respond and then progress whilst still on treatment) [125]. Since anti-angiogenic therapy targets tumour cells indirectly by acting on tumour blood vessels, mechanisms that determine response and resistance are likely to stem from a complex interaction between tumour cells and stroma.

**Fig. 3:**
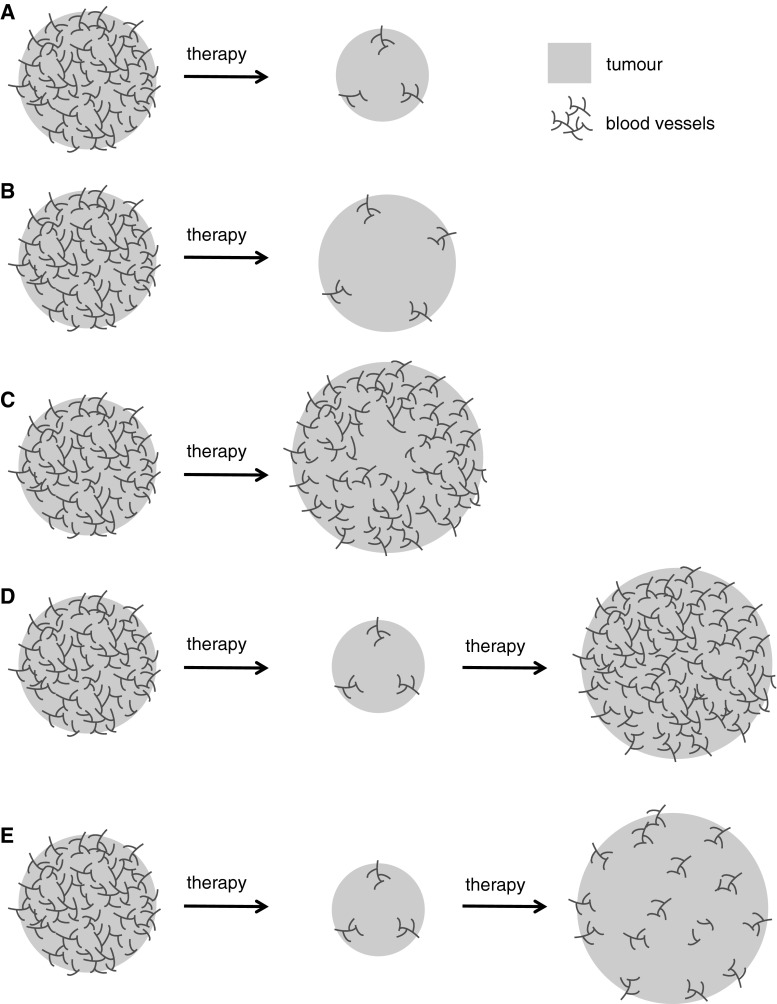
Response and resistance to anti-angiogenic therapy. Tumours may respond initially to anti-angiogenic therapy in different ways. **a** Therapy results in a strong vascular response (a significant reduction in the amount of perfused tumour vessels) and significant tumour shrinkage. **b** Therapy results in a strong vascular response, but only stabilisation of disease is achieved. **c** Therapy results in a poor vascular response (minimal reduction in the amount of perfused tumour vessels) and tumour stabilises or progresses. **d**, **e** After a period of response, acquired resistance can occur. This may be due to the activation of alternative angiogenic pathways (**d**) or because tumour cells adapt to the lack of a vascular supply via various potential mechanisms (**e**)

Insight into this tumour-stromal relationship in the setting of intrinsic resistance can be gained from studies in mRCC patients, which examined both change in tumour blood flow and change in lesion size in clinically detectable tumours upon treatment with single agent anti-angiogenic therapy [121–123]. In some cases, a strong vascular response may be observed, which is accompanied by significant tumour shrinkage (Fig. 3a) [121–123]. Tumours undergoing this type of response probably fulfil two important conditions: (a) the growth and survival of the vasculature is very sensitive to the agent, and (b) tumour cell survival is highly dependent on the vascular supply. In the second instance, despite a strong vascular response, tumour growth is only stabilised (Fig. 3b) [121–123]. In this scenario, tumour cells may be adapted to survive, despite a reduction in vascular supply. In the third instance, the targeted agent results in minimal or insignificant suppression of the tumour vascular supply, resulting in stabilisation of disease or tumour progression (Fig. 3c) [121–123]. In this scenario, the growth and survival of the vasculature is apparently poorly sensitive to the agent.

Longitudinal assessment of mRCC patients treated with these agents demonstrates that acquired resistance to therapy can also arise following a period of initial disease control [121–123]. Acquired resistance may conceivably occur because the tumour finds alternative means to drive tumour vascularisation which are insensitive to the therapy (Fig 3d) or because tumour cells become adapted so that they can grow despite the reduced vascular supply (Fig 3e) [123]. Evidence for specific cellular and molecular mechanisms that may underlie intrinsic or acquired resistance to anti-angiogenic therapy are discussed below.

### Heterogeneity of tumour blood vessels

**Fig. 4:**
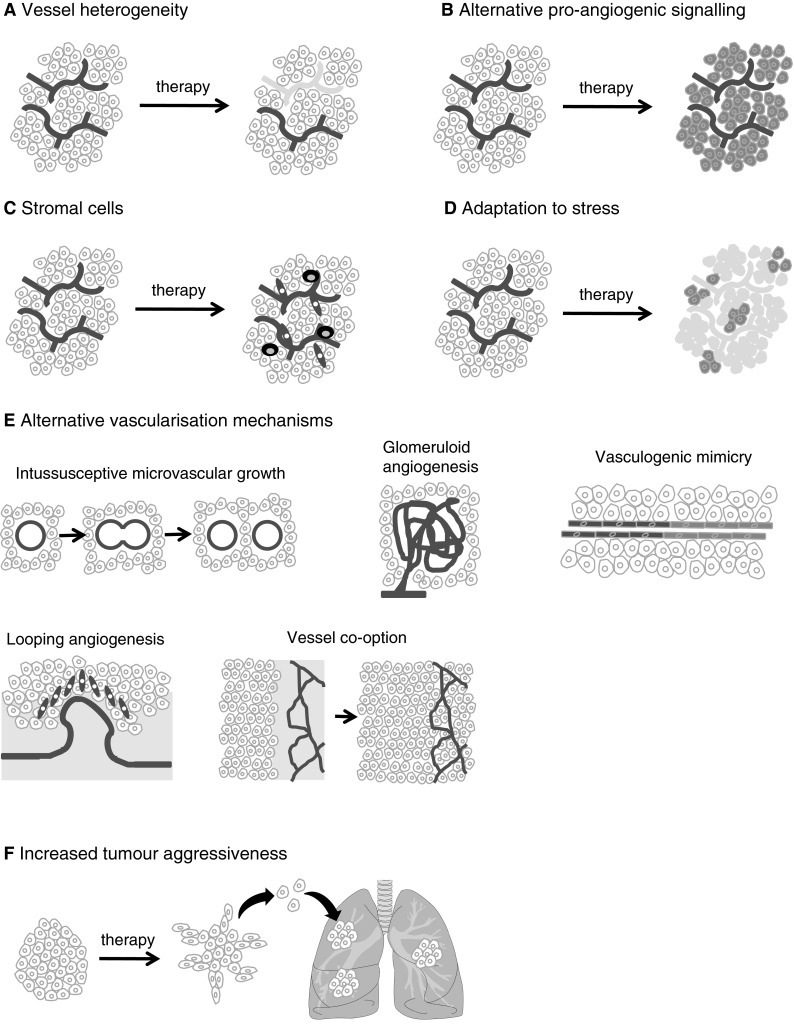
Potential mechanisms involved in resistance to VEGF-targeted therapy. **a** Tumours present with a mixture of therapy-sensitive and therapy-insensitive vessels. The top vessel is destroyed by the therapy (depicted in grey), whilst the bottom one remains (depicted in red). **b** Alternative signalling pathways can regulate the sensitivity of vessels to therapy. In the panel, the tumour cells (in blue) have up-regulated an alternative pro-angiogenic growth factor in order to drive blood vessel growth and survival. **c** Stromal cells, such as immature myeloid cells (black) or fibroblasts (green) infiltrate the tumour and mediate resistance either by releasing pro-angiogenic growth factors or by physically incorporating into vessels. **d** Tumour cells can survive conditions of stress. Some tumour cells (depicted in blue) have survived the loss of a vascular supply, because they are adapted to survive conditions of hypoxia or nutrient shortage. **e** Tumours may use alternative mechanisms of vascularisation besides sprouting angiogenesis. In intussusceptive microvascular growth new vessels are generated by the fission of existing vessels. Glomeruloid angiogenesis is characterised by tight nests of vessels that resmemble the renal glomerulus. In vasculogenic mimicry, tumour cells directly form vascular channels (blue cells) that are perfused via connection to the host vasculature (red cells). In looping angiogenesis, contractile myofibroblasts (green) pull host vessels out of the normal surrounding tissue (pink region). In vessel co-option tumour cells engulf host vessels in the normal surrounding tissue (pink region) as the tumour invades. **f** Increased tumour aggressiveness i.e. therapy causes tumour to become more invasive and/or accelerates the growth of metastases

The tumour vasculature is heterogeneous with respect to its response to anti-angiogenic therapy, with some vessels being sensitive whilst others are resistant (Fig. 4a). In preclinical studies, VEGF-targeted therapy suppresses the growth of newly formed tumour vessels, but is less effective against more established tumour vasculature [125–127]. The prevailing explanation is that nascent tumour blood vessels are dependent on VEGF, but eventually lose this dependence due to a process of ‘vessel maturation.’ Newly formed tumour vessels may mature via different routes, leading to the formation of at least six different types of tumour blood vessel, which vary in their sensitivity to VEGF-targeted therapy [126]. One aspect of vessel maturation is the recruitment of pericytes to tumour vessels, mediated by platelet-derived growth factors (PDGFs). It has been demonstrated that inhibition of PDGF-mediated pericyte recruitment improves the efficacy of VEGF-targeted therapy [128, 129]. Of interest, many clinically approved anti-angiogenic TKIs are potent inhibitors of both VEGF and PDGF receptors (e.g. sunitinib, sorafenib, pazopanib) and may therefore target pericyte recruitment. However, paradoxically, in xenograft models TKIs have been shown to result in either decreased or increased pericyte coverage, dependent on the study [130–133]. Therefore, whilst mature tumour vessels may be resistant to VEGF-targeted therapy, it is not currently clear how these tumour vessels can be effectively targeted.

### Alternative pro-angiogenic signalling pathways

Other pro-angiogenic signalling pathways can stimulate blood vessel growth and blood vessel survival even when the VEGF-pathway is blocked (Fig. 4b). Pre-clinical studies have identified numerous candidates including angiopoietins [129], Bv8; Bombina variagata peptide 8 [134], EGF; epidermal growth factor [135], the Delta-Notch pathway [136], FGF1 and FGF2; fibroblast growth factors 1 and 2 [137, 138], HGF; hepatocyte growth factor [139], IL-8; interleukin 8, [140], PDGF-C; platelet derived growth factor-C [141, 142] and PLGF; placental growth factor [26]. Most of these studies also show that co-targeting of VEGF and the candidate factor improves therapeutic response. Therefore, therapies that target signalling by multiple pro-angiogenic growth factors may be necessary to achieve efficient and durable suppression of tumour angiogenesis and tumour growth. There is also clinical evidence showing that circulating levels of certain pro-angiogenic factors, including FGF2, HGF, PLGF and SDF-1α can become elevated in patients just prior to progression on anti-angiogenic therapy, providing potential evidence that these factors are indeed related to the development of acquired resistance [143, 144].

However, the concept that these alternative growth factor and cytokine signalling pathways mediate resistance to anti-angiogenic therapy has yet to be truly validated clinically. The majority of TKIs used to treat patients (including brivanib, cediranib, dovitinib, sunitinib, sorafenib, vatalanib and many others) are multitargeted in nature and can suppress the signalling of multiple pro-angiogenic signalling pathways, including VEGF, FGF and PDGF. And yet, despite this, tumours have been shown to progress through treatment with these agents in many indications, including metastatic breast cancer [44–47], glioblastoma [75], hepatocellular carcinoma [145, 146] and mRCC [147]. This is in contrast to preclinical studies demonstrating a role for alternative growth factor signalling pathways and questions the relevance of alternative pro-angiogenic growth factors in mediating resistance to anti-angiogenic therapy in patients.

### Infiltrating stromal cells

It is now well established that tumours are a community composed of both transformed tumour cells and distinct stromal cell types. These stromal cells include fibroblasts and many different kinds of immune cell (such as lymphocytes, granulocytes and macrophages) as well as the cells that make up the vasculature (endothelial cells and pericytes). The roles played by these different stromal cell types in tumour progression have been extensively reviewed [148–153]. Importantly, the tumour stroma can promote tumour progression and therapy resistance, including resistance to anti-angiogenic therapies [154–157]. Preclinical studies have demonstrated that infiltration of tumours by various stromal cell types, including immature myeloid cells [158, 159], endothelial progenitor cells [160] or fibroblasts [141] can all mediate resistance to VEGF-targeted agents in preclinical models (Fig. 4c). Although the precise mechanisms through which these cells mediate resistance to anti-angiogenic therapy is not completely clear, they may promote the survival of tumour vessels and/or tumour cells through the secretion of growth factors such as BV8, in the case of immature myeloid cells [134], or PDGF-C, in the case of fibroblasts [141]. Alternatively, there is evidence that immature myeloid cells and endothelial progenitor cells may promote resistance to therapy by physically incorporating into tumour vessels [161–163].

### Adaptation of tumour cells to conditions of stress

Inhibition of tumour vascularisation should reduce the supply of oxygen and nutrients to tumours and slow tumour growth. However, preclinical work shows that tumour cells can be adapted to survive, even when the vascular supply is significantly reduced. These survival mechanisms include a reduced propensity for certain tumour cells to die under conditions of stress and may be driven by genetic aberrations such as loss of p53 function [164, 165]. Tumours treated with anti-angiogenic agents may also adapt to survive under conditions of nutrient withdrawal and hypoxia, by adapting their metabolism or through autophagy [130, 166–170]. Pre-adaptation or reactive adaptation to stress may therefore play a key role in determining whether tumours respond to VEGF-targeted therapies (Fig. 4d) [169, 171].

### Alternative mechanisms of tumour vascularisation

Despite a prevailing dogma that tumours utilise mainly VEGF-dependent sprouting angiogenesis (Fig. 1), it is now apparent that tumour vascularisation may occur via diverse mechanisms, including intussusceptive microvascular growth (IMG), glomeruloid angiogenesis, vasculogenic mimicry, looping angiogenesis and vessel co-option [3, 172, 173]. IMG is a process that generates two new vessels via the fission of an existing vessel (Fig. 4e). It has been observed in human primary melanoma and glioblastoma [174, 175]. Glomeruloid angiogenesis results in tight nests of tumour vessels known as a glomeruloid bodies (Fig. 4e). Glomeruloid bodies have been reported in a wide range of malignancies, including glioblastoma, melanoma, breast, endometrial and prostate cancer [176]. In vasculogenic mimicry, tumour cells organise into vessel-like structures that are perfused via connection to the host vasculature (Fig. 4e). It has been reported in many human cancers, including melanoma, breast, ovarian, prostate and sarcoma [177]. Recent pre-clinical studies suggest that tumour stem cells can directly differentiate into endothelial cells or pericytes, which may be a mechanism for vasculogenic mimicry [178–180]. In looping angiogenesis, vessels are extracted from normal surrounding tissue by the action of contractile myofibroblasts [181] (Fig. 4e). Although only well-characterised in wound healing, tumours might conceivably also utilise looping angiogenesis [181]. In vessel co-option, tumours recruit existing local blood vessels as they invade into surrounding host tissue (Fig. 4e). Analysis of human cancers reveals vessel co-option in glioblastoma [182, 183], adenocarcinoma of the lung [184, 185] cutaneous melanoma [186], lung metastases of breast and renal cancer [187–189], liver metastases of colorectal and breast cancer [190, 191] and brain metastases of lung and breast cancer [192].

Importantly, these alternative mechanisms of angiogenesis may be VEGF-independent and therefore capable of mediating tumour vascularisation despite VEGF-inhibition. For example, intussusceptive microvascular growth was demonstrated as a mechanism via which tumours can escape the effects of TKIs in a preclinical model of mammary carcinoma [193]. Moreover, preclinical and clinical data show that tumours in the brain can become more infiltrative when the VEGF pathway is inhibited, which may facilitate vessel co-option [54, 182, 183, 194–198]. However, despite these data, we have very little understanding of the molecular mechanisms that control these alternative mechanisms of tumour vascularisation.

### Increased tumour aggressiveness

Some pre-clinical studies report that VEGF-targeted therapy can promote increased tumour invasion and metastasis (Fig. 3f) [196, 199–201]. Paez-ribes et al. [196] and Sennino et al. [200] demonstrated in a genetically engineered mouse model (GEMM) of PNET (RIPTag mice), that pharmacological inhibition of the VEGF pathway (VEGF receptor inhibitory antibody or sunitinib) suppressed the growth of the primary tumour. However, the treated tumours became more invasive and showed an increased incidence of liver and lung metastasis, compared to vehicle controls. Ebos et al. [202] demonstrated that sunitinib can suppress tumour growth when breast cancer or melanoma cells are implanted into the primary site (i.e. mammary fat pad or skin, respectively). However, administration of sunitinib either prior to, or after, resection of the primary tumour increased the incidence of metastasis and led to a shortening of overall survival, compared to vehicle controls [202]. In the same study, treatment of mice with sunitinib prior to, or after, intravenous injection of tumour cells also promoted the growth of metastases and shortened overall survival, compared to vehicle controls [202]. These data imply that VEGF-targeted therapies could accelerate tumour progression when used in the metastatic, adjuvant or neoadjuvant setting.

Although these results are alarming, follow-up pre-clinical studies from other laboratories challenge some of these findings [130, 203, 204]. Chung et al. [204] treated four different GEMMs with a VEGF inhibitory antibody and failed to find any evidence that treatment increased the incidence of metastasis. However, they did observe increased invasion and metastasis in a GEMM of PNET treated with sunitinib [204]. Two further studies examined more closely the ability of sunitinib to accelerate metastasis in mice. Both Welti et al. [130] and Singh et al. [203] showed that administration of sunitinib to mice, prior to intravenous injection of tumour cells, only promotes metastasis when sunitinib is administered at very high doses, but not when lower, clinically relevant doses are utilised. In addition, Welti et al. [130] showed that although sunitinib treatment is associated with a worse prognosis in a model of metastatic breast cancer, sunitinib treatment was able to prolong survival in a model of metastatic renal cancer.

Is there evidence that anti-angiogenic therapy can promote tumour aggressiveness in patients? A retrospective analysis of mRCC patients treated with sunitinib found no evidence of accelerated tumour growth, suggesting that sunitinib does not accelerate tumour growth in advanced renal cancer [205]. However, there are some reports of rapid tumour progression in individuals with mRCC after withdrawing anti-angiogenic therapy, a phenomenon sometimes referred to as ‘flare-up’ [54, 89, 90, 206]. It has been shown that, upon withdrawal of anti-angiogenic therapy, the tumour vasculature can rapidly re-grow [87, 88]. Moreover, a recent neoadjuvant study of sunitinib and pazopanib in mRCC demonstrated a paradoxical increase in Ki67 and tumour grade in the primary tumour after treatment [207]. These findings might provide some clues to the source of the flare-up phenomenon, but the precise mechanisms are as yet unclear.

The influence of bevacizumab treatment withdrawal has also been assessed in patients. A retrospective analysis of five large studies (which included patients with mRCC, metastatic pancreatic cancer, metastatic breast cancer and metastatic colorectal cancer) found no evidence that discontinuation of bevacizumab treatment lead to accelerated disease progression compared to placebo controls [208]. Some data examining this question in the adjuvant setting are also available. Analysis of the NSABP-C08 trial of adjuvant bevacizumab in colorectal cancer failed to provide evidence for a detrimental effect of exposure to bevacizumab [56]. However, data from the AVANT trial of adjuvant bevacizumab in colorectal cancer did find evidence that treatment with bevacizumab was associated with a detrimental effect: a higher incidence of relapses and deaths due to disease progression was observed in the bevacizumab treated patients [57]. It has been proposed that the disappointing results obtained in the adjuvant setting with bevacizumab could be explained by an adverse effect of bevacizumab on tumour biology: increased aggressiveness of the cancer [54].

There is one setting in which the induction of a more invasive tumour phenotype upon treatment with anti-angiogenic therapy is relatively undisputed. Glioblastomas have been observed to adopt a more infiltrative tumour growth pattern upon treatment with VEGF-targeted therapy [182, 183, 209]. Interestingly, it seems plausible that this invasive process can contribute to resistance to anti-angiogenic therapy by allowing vessel co-option to occur [195].

In conclusion, there is conflicting evidence for the relevance of increased tumour aggressiveness in response to anti-angiogenic therapy and this persists as a controversial area [54, 210, 211]. However, taken together, the available data suggest that the ability of VEGF-pathway targeted agents to promote tumour aggressiveness is influenced by several factors, including cancer type, the stage of disease being treated (neoadjuvant, adjuvant or metastatic) the nature of the anti-angiogenic agent administered, the dose of agent that the recipient is exposed to and the physiology of the individual patient.

The mechanisms that underlie the increased invasiveness and increased metastasis observed in some studies of VEGF-targeted therapy are the subject of ongoing investigation. Several studies have demonstrated that VEGF-targeted therapy can cause tumour cells to undergo an epithelial-to-mesenchymal transition, which could promote increased invasion and metastasis [200, 201, 212, 213]. Activation of the MET receptor has been implicated in the process of increased invasion and metastasis observed upon VEGF-targeted therapy in preclinical models, and simultaneous inhibition of VEGF and MET signalling was shown to suppress the increased invasion and metastasis observed in preclinical models of PNET and glioblastoma [199–201].

Another possible causative factor in the enhanced metastasis observed in angiogenesis inhibitor treated mice is a drug-induced change in circulating factors. For example, it has been shown that TKIs in particular can induce a significant change in a number of circulating factors implicated in tumour progression including G-CSF, SDF-1α and osteopontin [214]. A change in levels of these factors could potentially contribute to tumour progression at distant sites. In support of this concept, a recent study showed that changes in circulating levels of interleukin-12b were required for the enhanced metastasis observed upon sorafenib treatment in a preclinical model of hepatocellular carcinoma [215].

It is known that the integrity of the vasculature is important in controlling metastasis [216, 217]. Therefore, another possible mechanism could be that VEGF-targeted therapies damage the vasculature, leading to enhanced tumour cell extravasation at the primary site or increased seeding at the metastatic site. There is some direct evidence in preclinical models that TKIs may promote metastasis by damaging the integrity of the vasculature [130, 199, 204].

Despite these data, more work is required to understand in which settings increased aggressiveness may be relevant and how it occurs at the mechanistic level. It remains to be seen whether combination strategies designed to inhibit both angiogenesis and invasion/metastasis will be necessary to achieve a better therapeutic index in patients.

### Signalling by VEGF in different cell types

Beyond its role in stimulating angiogenesis in endothelial cells, it is now apparent that VEGF can play a signalling role in many other cell types. These include: endothelial cells of the normal vasculature [218], dendritic cells [219], myeloid cells [220], neurons [221], pericytes [222] and tumour cells [201, 212, 223–228]. Identification of these additional physiological and pathophysiological roles for VEGF has led to some surprising observations. For example, inhibition of VEGF in the normal vasculature may be the cause of certain side effects seen in patients treated with VEGF-targeted agents, such as hypertension [81], whilst suppression of VEGF signalling in myeloid cells was shown to accelerate tumourigenesis in mice [220]. This latter phenomenon may be another mechanism leading to increased aggressiveness in cancers treated with anti-angiogenic therapy.

In addition, there are numerous studies documenting a role for VEGF signalling in tumour cells, but the data are conflicting. Several studies have shown that cancer cell lines can express VEGFR1 or VEGFR2 and that signalling through these receptors in cancer cells can promote events associated with tumour progression, including cancer cell survival, proliferation, invasion or metastasis [224–229]. Based on these data it has been proposed that inhibition of VEGF signalling in tumour cells may, at least in part, be mediated by direct activity against tumour cells [4]. In contrast, more recent preclinical studies have shown that inhibition of VEGF signalling in CRC and glioblastoma cells made these cells more invasive [201, 212]. These latter data suggest that, in fact, targeting VEGF signalling in cancer cells may actually be deleterious. Further studies are warranted to untangle this dichotomy.

### Interactions between VEGF receptors and other cell surface receptors

The VEGF signalling system in mammals is complex and consists of five related ligands, VEGF-A, VEGF-B, VEGF-C, VEGF-D and PLGF that bind with different specificities to three receptor tyrosine kinases, VEGFR1, VEGFR2 and VEGFR3. In addition, several co-receptors have been identified, including heparin sulphate proteoglycans, neuropilin 1 (NRP1), neuropilin 2 and CD146. Moreover, VEGF receptors can cross-talk with additional cell surface molecules, including integrins and other growth factor receptors. The biology of this complex signalling system has been extensively reviewed [8, 230–232]. Here we will focus on some selected studies that examined the relevance of these interactions in determining response or resistance to VEGF-targeted therapies in cancer.

PLGF is overexpressed in many cancers and signals by binding to VEGFR1 [233]. Combined inhibition of VEGF and PLGF was shown to be more effective at suppressing primary tumour growth than VEGF inhibition alone in several preclinical models [26, 234]. However, these results were challenged in a publication showing that, although inhibition of PLGF can suppress metastatic spread, it had no effect on the growth of primary tumours [235]. Co-receptors for VEGFR2, including NRP1 and CD146, may act to amplify signal transduction through VEGFR2, leading to an increased angiogenic response [233]. Combined inhibition of NRP1 and VEGF [236], or CD146 and VEGF [237], were both shown to be more effective than inhibition of VEGF alone in preclinical primary tumour models.

VEGFR2 can also form direct complexes with other receptor tyrosine kinases. For example, stimulation of vascular smooth muscle cells with VEGF promotes the formation of a complex between VEGFR2 and the receptor tyrosine kinase PDGF-Rβ [222]. This results in suppression of PDGF-Rβ signalling and decreased pericyte coverage in tumours [222] and may explain the observation that, in some experimental systems, inhibition of VEGF signalling leads to increased pericyte coverage of tumour vessels and increased maturation/normalisation of the tumour vasculature [238]. Moreover, in glioblastoma cells, VEGF stimulates the formation of a complex between VEGFR2 and the receptor tyrosine kinase, MET, which results in suppression of MET signalling and reduced tumour cell invasion [201]. As a consequence of this, inhibition of VEGF was shown to release MET from this inhibitory mechanism and allow for increased tumour invasion [201]. Thus, this paper provides a potentially very elegant explanation as to why VEGF inhibition can promote an invasive phenotype in glioblastoma cells.

Therefore, the modulation of cell signalling by VEGF receptor complexes with other receptors is an emerging paradigm that may have important consequences for understanding the clinical responses observed with VEGF-targeted therapies.

## Future directions for anti-angiogenic therapy

Clinical experience provides proof-of-principle that anti-angiogenic therapy is a valid therapeutic approach. The full potential of this strategy is, however, yet to be realised. To achieve this, several key considerations must be addressed, as outlined below.

### Understanding the vascular biology of different primary cancers and their metastases

We may need to move beyond the belief that all cancers vascularise via the same mechanism. Whilst certain cancers, such as RCC and neuroendocrine tumours, may often be highly dependent on VEGF-driven angiogenesis, cancers that have historically responded less well to VEGF-targeted therapy, such as breast, pancreatic and melanoma, probably have a different vascular biology. Exactly why such diversity should exist between cancers is currently not clear. Tumour evolution is most likely an important factor. For example, given that inactivation of the Von Hippel-Lindau (VHL) gene is a frequent early event in renal cancer that results in elevated expression of VEGF [239], it is perhaps not surprising that the aetiology of these tumours is strongly coupled with a dependence on VEGF-driven angiogenesis. However, in other cancers where VHL inactivation is not prevalent, VEGF-driven angiogenesis may be just one of several tumour vascularisation pathways that the cancer can evolve to utilise. Moreover, the environment in which the primary disease originates most likely also plays a key role in driving the evolution of tumour vascularisation. The vasculature is not a homogenous entity: considerable heterogeneity of form and function is observed between different organs [240]. As different types of primary tumours evolve in different organs (e.g. brain, breast, colon, skin, kidney, liver, lung, pancreas, etc.) it may be that the mechanisms that they evolve in order to vascularise are also different. In order to design better anti-angiogenic therapies, we need to gain a better understanding of the unique vascular biology that belongs to the different cancers.

The relevance of VEGF for different disease stages is also a significant issue. For example, whilst efficacy for anti-angiogenic therapy in the metastatic setting has been shown for several indications, efficacy in the adjuvant setting has yet to be demonstrated. Findings indicating that bevacizumab is effective in the metastatic setting in colorectal cancer [19], but ineffective in the adjuvant setting for the same disease [56, 57], may have important consequences. Many trials of anti-angiogenic agents in the adjuvant setting are currently underway. Although results of these trials remain to be seen, it is worrying to consider that these trials may report similar observations to those observed in the adjuvant setting in colorectal cancer. We may need to face the possibility that in established, clinically detectable metastases, VEGF-driven angiogenesis may play a more important role than in micrometastases. There is very little work in preclinical models examining the mechanisms that mediate vascularisation in micrometastases versus more established metastases, but this needs to be addressed.

Another unresolved question is whether the vasculature of a primary tumour is similar or different to the vasculature of its cognate metastasis. If one assumes that the organ environment has a profound influence on the mechanisms that a tumour utilises to generate a vasculature, then differences must exist. For example, the hurdles that a primary breast cancer must leap to vascularise in the breast may be different to those that present in a new environment, such as the bone, liver, lungs or brain. In support of this, the colonisation of new organ environments during metastasis is thought to be inefficient [241]. One reason for this may be that tumours must ‘re-educate’ in order to vascularise in the new environment. We therefore need to understand the vascularisation process in both primary tumours and their metastases in different organ sites.

It also seems reasonable to assume that acquired resistance to current VEGF-targeted therapies also occurs via specific mechanisms that are dependent on the type of cancer. For example, new vessel growth driven by alternative pro-angiogenic growth factors, such as FGF2, HGF or IL-8, may drive acquired resistance to TKIs in RCC or neuroendocrine tumours [137, 138, 140, 144]. Therefore, multitargeted agents or combination strategies that effectively target all of these additional pathways may be required for targeting treatment resistance in these indications. In contrast, acquired resistance in glioblastoma may occur due to increased tumour invasion and vessel co-option [182, 183, 195, 198, 201]. Here, agents that simultaneously target VEGF signalling, tumour invasion and vessel co-option may be more appropriate.

### The role of tumour heterogeneity

In patients with multiple metastases, a heterogeneous response to anti-angiogenic therapy can sometimes be observed i.e. some lesions may respond whilst other lesions in the same patient can progress [123]. This is challenging for optimal patient management and continuation of therapy, and may herald early treatment failure. Although the source of this heterogeneity is poorly understood, one explanation could be that diverse tumour vascular biology can exist in a patient. For example, histopathological studies on human lung and liver demonstrate that tumours present in these sites display significant intra- and inter-tumour heterogeneity, utilising either angiogenesis or vessel co-option to gain access to a vascular supply [173, 184, 185, 187, 190, 191, 242, 243]. This suggests that, within the same tumour and between different tumours in the same patient, more than one mechanism to become vascularised can be utilised at any particular time. Moreover, comprehensive genomic analysis of tumours reveals significant genetic intra- and inter-tumour heterogeneity [244]. Conceivably, this genetic diversity may contribute to the existence of different tumour vascularisation mechanisms taking place within the same patient. Understanding how this heterogeneity occurs and how to target it effectively is a key goal, not just for anti-angiogenic therapy, but for all cancer therapeutics [244, 245].

### Preclinical experiments that translate to clinic

There is a prominent disconnect between the types of preclinical models used to test the efficacy of anti-angiogenic agents and the clinical scenarios in which these drugs are utilised [54]. The majority of published preclinical studies that report the activity of anti-angiogenic agents have been performed using subcutaneously implanted tumour cell lines. Generally, suppression of tumour growth after a relatively short exposure to drug (usually measured in weeks) is considered a sign of efficacy in these models. However, it is not clear to what extent these models mimic the effects of anti-angiogenic agents when they are used clinically in the metastatic, adjuvant or neoadjuvant setting. Moreover, very few studies use survival as an endpoint. In support of the need for refined models, recent preclinical studies clearly demonstrated that whilst anti-angiogenic therapies can be effective at controlling tumour growth in models of the primary disease, the same therapies were not effective in models of the adjuvant or metastatic treatment setting [202, 246]. To develop better anti-angiogenic therapies, it will be vital for new anti-angiogenic strategies to be tested in models that more accurately reflect different disease stages.

In addition, there are a growing number of studies demonstrating that resistance to VEGF-targeted agents might be overcome by targeting a second pathway. This includes targeting additional pro-angiogenic signalling pathways [26, 137–141, 236, 237, 247, 248] or by targeting compensatory metabolic or pro-invasive responses in tumour cells [166, 168, 170, 200, 201]. These studies are vital and should allow the design of rationale combination strategies that could be tested in the clinic. However, there are several practical problems associated with this, including finding targets that are easily druggable and selecting combinations that have an acceptable toxicity profile [249]. A consideration of these practicalities at the preclinical phase may accelerate the selection of new strategies that can be practically and rapidly translated to the clinic.

### Development of appropriate predictive biomarkers

As we have seen, the biology determining response and resistance to anti-angiogenic therapy is complex. It is perhaps therefore unsurprising that predictive biomarkers for this class of agent remain elusive. To identify which patients will benefit from these therapies, mechanism-driven biomarkers are required that can account for the dynamic and complex underlying biology. Importantly, as more and more promising biomarkers are uncovered, a further challenge will be to standardise methods of biomarker assessment across centres so that they can be validated prospectively and, eventually, utilised routinely.

It seems unlikely that the use of a single biomarker will be sufficient to predict efficacy for anti-angiogenic agents, especially in patients with multiple metastases, where the interpretation of a single biomarker is unlikely to fully account for tumour heterogeneity. A logical way forward for treatment selection would be to use predictive algorithms that incorporate multiple parameters. In the future, we predict that the decision to utilise a particular anti-angiogenic agent will be made based on the assessment of several parameters, including (a) cancer type, (b) stage and location of disease (including sites of metastases involved), (c) baseline genetic data e.g. germline SNPs, (d) circulating markers acquired at baseline and during therapy, and (e) functional imaging data acquired both at baseline and during therapy. Moreover, in a world where multiple targeted agents are now potentially available for tailored treatment, the decision to use anti-angiogenic therapy will need to be weighed against the use of other potentially effective treatment options for each patient.

### Alternative approaches for targeting the tumour vasculature

Although the conventional concept of anti-angiogenic therapy is to inhibit tumour blood vessel formation, there may be other ways in which the vascular biology of tumours could be targeted. Of course, one long-standing hypothesis is that therapies should be designed to normalise the tumour vasculature in order to improve the delivery of chemotherapy [71, 72, 238]. This might be particularly pertinent in poorly vascularised cancers such as pancreatic adenocarcinoma where improved delivery of chemotherapy could be beneficial [250]. Moreover, vascular normalisation may have additional beneficial effects for controlling oedema or tumour oxygenation [74, 75]. In addition, it is now known that blood vessels are not merely passive conduits for the delivery of oxygen and nutrients. Beyond this, they can secrete specific ‘angiocrine factors’ that can control embryonic development, tissue regeneration and tumour growth in a perfusion-independent manner [251]. Furthermore, two recent studies showed that endothelial cells can secrete specific ligands that induce chemoresistance in tumour cells [252, 253]. These studies reflect a growing paradigm that the tumour stroma plays an important role in therapy resistance [150, 154, 156, 157].

Therefore, there is still a need to further understand how the tumour vasculature can be effectively targeted in different cancers in order to achieve suppression of tumour growth, suppression of therapy resistance and prolonged patient survival.

## Conclusions

Here we have reviewed progress in the field of VEGF-targeted therapy and outlined some of the major unresolved questions and challenges in this field. Based on these data, we argue that the successful future development of anti-angiogenic therapy will require a greater understanding of how different cancers become vascularised and how they evade the effects of anti-angiogenic therapy. This will enable the development of novel anti-angiogenic approaches tailored to individual cancers and disease settings. Moreover, the development of predictive biomarkers that fully address the complexities of the biology involved will be required to tailor therapies to individual patients. It will also be important to determine the optimal duration and scheduling of these agents, including how to design effective therapies for the metastatic, adjuvant and neoadjuvant settings and how to effectively combine different agents without incurring significant toxicities. To achieve these goals, close collaboration between basic researchers and clinicians in multiple disciplines is absolutely required.
